# Effects of the COVID-19 pandemic and lockdown on the mental and physical health of adults with Prader-Willi syndrome

**DOI:** 10.1186/s13023-021-01833-1

**Published:** 2021-05-05

**Authors:** Helena Mosbah, Muriel Coupaye, Flavien Jacques, Maithé Tauber, Karine Clément, Jean-Michel Oppert, Christine Poitou

**Affiliations:** 1 Assistance Publique-Hôpitaux de Paris, Centre de Référence Maladies Rares (PRADORT, Syndrome de Prader-Willi Et Autres Formes Rares D’Obésité Avec Troubles du Comportement Alimentaire), Service de Nutrition, Groupe Hospitalier Pitié-Salpêtrière, Hôpital Pitié-Salpêtrière, Sorbonne Université, 91 bd de l’Hôpital, Bâtiment E3M, 75013 Paris, France; 2 INSERM, Nutrition et Obésité: Approches Systémiques «NutriOmics», Sorbonne Université, Paris, France; 3 Centre de Référence du Syndrome de Prader-Willi, Service d’Endocrinologie, Obésités, Maladies Osseuses Génétique et Gynécologie Médicale, Hôpital des Enfants, Toulouse, France

**Keywords:** Prader-Willi syndrome, Intellectual disability, Genetic obesity, COVID-19, Lockdown, Physical activity, Eating behavior

## Abstract

**Background:**

Prader-Willi syndrome (PWS) is a neurodevelopmental disorder with hypothalamic dysfunction leading to obesity and behavioral disabilities, including eating disorders (EDs). We evaluated the effects of the COVID-19 infection and lockdown on mental and physical health in PWS. At the end of April, 85 adults with PWS completed a self-administered questionnaire, including lockdown conditions, physical activity (PA), ED, and medical and behavioral outcomes. Body weight was measured at home and self-reported.

**Results:**

Patients (52.9% women, 44.8% disomic) were assessed, with a mean age of 28.05 ± 8.73 years and body mass index (BMI) of 36.76 ± 10.74 kg/m^2^. Seventy percent lived in the Paris region (France) and were confined with their parents. The mean weight change was 0.96 ± 3.28 kg. We compared patients showing weight loss (n = 39, − 3.30 ± 2.93 kg) to patients showing weight gain (n = 22, + 2.35 ± 1.54 kg): the BMI was lower (34.60 ± 9.18 versus 40.45 ± 9.45 kg/m^2^, *p* = 0.02), PA increased (25.6% versus 4.5%, *p* = 0.04), and EDs improved (51.3% versus 13.6%, *p* = 0.005). Behavioral disorders increased for 12.9% of the cohort. Three individuals (3.5%) were diagnosed with non-severe COVID-19.

**Conclusion:**

Lockdown during the COVID-19 pandemic was associated with positive effects for most French adults with PWS, with weight loss probably associated with a more favourable environment during this period. We observed no severe forms of COVID-19.

## Background

Prader-Willi Syndrome is the most frequent etiology of syndromic obesity of which the prevalence is approximately 1/20,000 newborns. This genetic syndrome results from the lack of expression of paternally inherited imprinted genes in chromosomal region 15q11.2-q13, leading to a neurodevelopmental disorder and hypothalamic dysfunction. Adults with PWS show difficulty in adapting to changes, emotional lability, hyperphagia, and food impulsivity. Outbursts may occur, caused by frustration over food or the misunderstanding of social situations [1]*.* Uncontrolled eating behavior can lead to severe obesity, associated with comorbidities (e.g., type 2 diabetes, hypertension). Early multidisciplinary care is required, with permanent supervision of eating practices and adapted physical activity (PA) [2].

During the COVID-19 pandemic, individuals with obesity [3, 4] and/or diabetes [5] were shown to be at higher risk of severe forms of COVID. Whether syndromic obesity, such as PWS, also represents a risk for severe forms of COVID-19 is unknown. Moreover, complete lockdown was implemented in France in mid-March 2020 to contain the spread of COVID-19. There was concern that individuals with PWS may experience decreased PA and poorer food control during the lockdown period, as observed for children with common obesity [6]*,* potentially worsening their condition*.* People with psychiatric disorders or/and intellectual disability were also shown to be particularly vulnerable, showing increased anger and impulsivity [7, 8]. The aim of this cross-sectional study was to assess changes in weight and mental and physical health in adults with PWS during the COVID-19 lockdown.

## Results

### Cohort characteristics (Table 1)

**Table 1:** General characteristics of Prader-Willi patients

*Patient characteristics*	
N	85
Age (years)	28.05 ± 8.73 [18.00–51.00]
Sex N (%)	Female 45 (52.9) Male 40 (47.1)
BMI (kg/m^2^)	36.76 ± 10.74 [19.60–68.00]
Diabetes N (%)	21 (24.7)
Hypertension N (%)	10 (11.8)
Dyslipidemia N (%)	16 (20.2)
Active smoking N (%)	7 (8.9)
*Genetics*	
del(15)(q11-q13) UPD(15)mat	Female 26 (30.5), male 21 (24.7) Female 19 (22.4), male 19 (22.4)
*Conditions of lockdown*	
Usual place of residence N (%)	Family home: 60 (70.6), health-care institution: 25 (29.4)
Geographic region N (%)	Greater Paris region: 59 (69.4)
Number of rooms/Number of habitants (except institutions)	1.42 ± 0.59 [0.50–3.67]
*Daily occupations*	
Usual daily activity N (%)	Work 27 (31.4) School 18 (20.9) Leisure activities 22 (25.6) No activity 19 (22.1)
*Physical activity*	
Change in physical activity N (%)	Increased 16 (18.8), decreased 57 (67.1), stable 12 (14.1)
Daily number of physical activities	1.54 ± 0.97 [0.00–4.00]
Type of physical activity (%)	Walking 39.1% Fitness 22.0% Exercise bike/treadmill 15.3% Gardening 11.2% Housekeeping 7.8% Outdoor games 4.6%
Change in time being sedentary N (%)	Increased 60 (75.0), decreased 5 (6.2) stable 15 (18.8)
*Sleep*	
Change in time spent sleeping N (%)	Increased 20 (24.1), decreased 5 (6.0), stable 58 (69.9)
*Weight/eating habits*	
Change in weight (kg and %)	kg: − 0.96 ± 3.28 [− 13.00; + 5.00] %: − 1.07 ± 3.26 [− 10.00; + 6.25]
Change in weight N (%)	Gain 22 (27.5), loss 39 (48.8), stable 19 (23.7)
Change in eating behavior N (%)	Worse 17 (20.7), improved 29 (35.4), stable 36 (43.9)
Aggressivity towards food N (%)	Yes 16 (20.0)
*Psychological impact*	
Difficulties during lockdown N (%)	Major 11 (12.9), minor 38 (44.7), none 36 (42.4)

Results are expressed as the means ± SD [range] for continuous variables and as frequencies and percentages for categorical variables. BMI: body mass index. del(15)(q11-q13): Deletion from the paternal origin in the 15q11-q13 chromosomic region. UPD(15)mat: Maternal UniParental Disomy of chromosome 15

Eighty-five patients with PWS were interviewed. Their mean age was 28.05 ± 8.73 years and their mean BMI 36.76 ± 10.74 kg/m^2^; 87.1% were overweight or obese. Fifty-five percent had a partial deletion from the paternal origin in the 15q11-13 chromosomal region (del(15)(q11-q13)). Forty-five percent had a maternal uniparental disomy of chromosome 15 (UPD(15)mat). Twenty-five percent of patients had diabetes, 11.8% had hypertension, 20.2% had dyslipidemia, 8.9% were active smokers. Concerning their hormonal system, 92.4% had hypogonadism, 50.6% had a growth hormone deficiency, 28.2% had hypothyroidism and 6.6% a corticotrop deficiency. Four patients were epileptic and 41.8% were under psychotropic drugs.

Seventy percent of patients were confined at their family home and 29.4% at their social healthcare institution. Seventy percent of patients were living in the greater Paris region and 10.6% (n = 9) in French regions where the prevalence of COVID-19 was high. The lockdown was total in 50.6% of cases, partial in 48.2% (one hour maximum per day outside of the house), and not respected in 1.2%. Seventy-seven percent of patients normally performed a daily activity that was completely stopped during lockdown.

### Weight change during lockdown (Table 2, Fig. 1)

**Table 2:** Characteristics of Prader-Willi patients according to weight change category

Characteristics	Weight loss	Weight gain	*p*-value
N	39	22	
Age (years)	28.95 ± 8.31 [18.00–51.00]	27.32 ± 9.98 [18.00–50.00]	0.20
BMI (kg/m^2^)	34.60 ± 9.18 [19.60–52.00]	40.45 ± 9.45 [22.51–58.60]	0.02
Change in weight (kg and %)	kg − 3.30 ± 2.93 % 3.68 ± 2.50	kg + 2.35 ± 1.54 % + 2.41 ± 1.57	0.0001
Sex N (%)	Female 22 (56.0), male 17 (44.0)	Female 11 (50.0), male 11 (50.0)	0.8
Type of residence N (%)	Institution 6 (15.4) Family home 33 (84.6)	Institution 2 (9.1) Family home 20 (91.1)	0.69
Usual daily activities N (%)	Yes 34 (87.2), no 5 (12.8)	Yes 15 (68.2), no 7 (31.8)	0.10
Change in physical activity N (%)	Increased 10 (25.6) No increase 29 (74.4)	Increased 1 (4.5) No increase 21 (95.5)	0.04
Change in time being sedentary (%)	Decreased 3 (7.7) No decrease 35 (92.3)	Decreased 1 (4.5) No decrease 21 (95.5)	> 0.99
Change in eating behavior (%)	Improvement 20 (51.3) No improvement 19 (48.7)	Improvement 3 (13.6) No improvement 19 (86.4)	0.005

Results are expressed as the means ± SD [range] for continuous variables and as frequencies and percentages for categorical variables. *BMI* body mass index

**Fig. 1:**
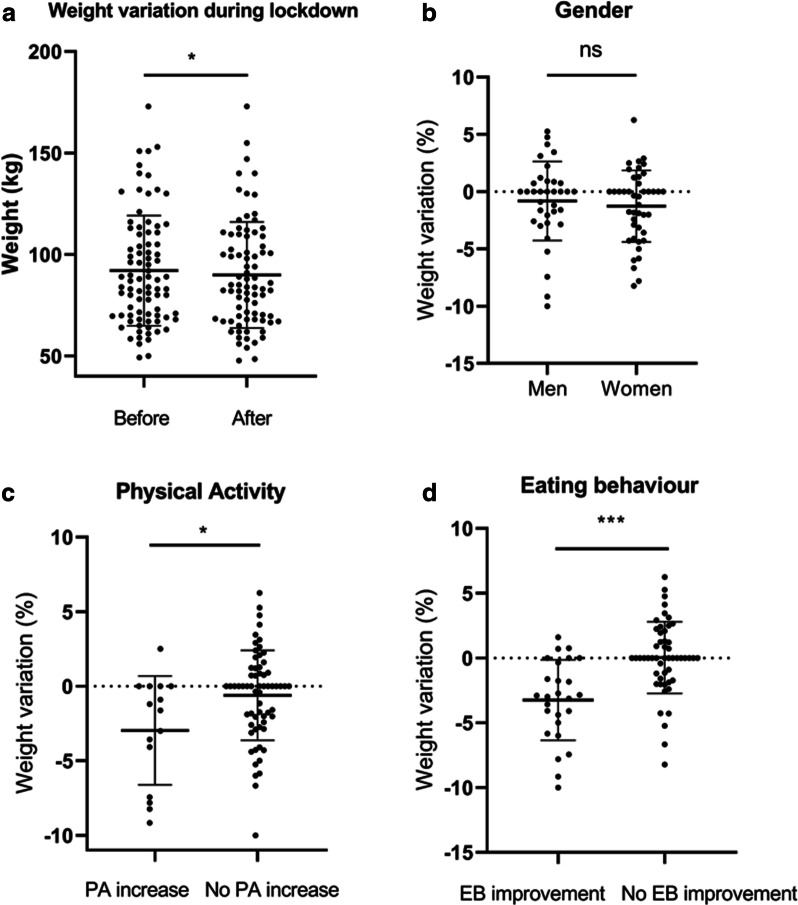
**a** Individual weights of members of the total PWS cohort (weight before lockdown, weight after lockdown, n = 85). **b** Change in weight (%) during the lockdown according to gender (n = 43 females and 37 males). **c** Change in weight (%) during the lockdown according to physical activity (increase n = 15, no increase n = 65). **d** Change in weight (%) according to eating behavior (improvement n = 26, no improvement n = 52). Results are expressed as the mean ± standard deviation, with individual values. ns = non-significant. *p < 0.05; ***p < 0.001. *EB* eating behavior, *PA* physical activity

During lockdown, 67.1% of patients reported a decrease in daily PA. The mean number of daily PAs was 1.54 ± 0.97/day [0.00–4.00]. Feeding behavior was reported to have improved for 35.4% of patients and did not change for 43.9%. Forty-nine percent of patients lost weight (mean ± SD: − 3.30 ± 2.93 kg), it was stable for 23.7%, and 27.5% gained weight (+ 2.35 ± 1.54 kg). Mean weight loss was 0.96 ± 3.28 kg (%: − 1.07 ± 3.26 [− 10.00; + 6.25], *p* = 0.01) (Fig. 1a).

Patients who lost weight had a lower BMI before the lockdown than those who gained weight (34.60 ± 9.18 versus 40.45 ± 9.45 kg/m^2^, *p* = 0.02). There were no differences between those two groups in terms of age or gender (Fig. 1b). Genetic subtype (deletion versus disomy) did not influence the weight evolution during the lockdown (*p* = 0.06). In terms of weight loss, there was no significant difference between rural (n = 23) and urban (n = 55) living conditions. Weight loss was more frequently observed for patients with increased PA (25.6% versus 4.5% *p* = 0.04) (Fig. 1c) and those with improved eating behavior (51.3% versus 13.6% *p* = 0.005) (Fig. 1d).

### Medical and mental health during the COVID-19 pandemic and lockdown

Three patients had symptoms of non-severe COVID-19 (3.5%), although 12.9% (n = 11) were living with relatives diagnosed with COVID-19. No confirmation with PCR was performed but a serological assay performed at the end of June confirmed seroconversion and SARS-Cov2 infection for the three patients. No hospitalization was required. Their age was 18–25 years, their BMI 21.20–48.80 kg/m^2^, and two had diabetes, with glycated hemoglobin between 6.6 and 8.2%.

Concerning behavioral issues, 11 patients (12.9%) displayed a recrudescence of behavioral disorders without a major effect on mean weight (+ 0.40 kg) and 10/11 presented more frequent outbursts, which required medical contact in two cases. Principal difficulties reported by caregivers were linked to exacerbations of anxiety and the ban on going outside.

Other medical issues among the PWS patients were the following: dermatological disorders (n = 7), traumatic accidents (n = 2), respiratory tract infections (n = 2), intestinal transit disorders (n = 2), epilepsy (n = 2), exacerbation of pain (n = 2), hypoglycemic events (n = 2), and dental disorders (n = 1).

## Discussion

The lockdown had a positive impact for most French adults with PWS. Nearly 50% lost weight, in parallel with improved lifestyle behaviors (eating habits, PA). No severe form of COVID-19 was noted, despite a high prevalence of obesity and diabetes. However, a small subgroup experienced major difficulties, with the recrudescence of behavioral disorders and anxiety.

In an Italian study, 20% of adult patients gained weight and reported an increase in the consumption of “comfort food” (chocolate, ice cream, desserts, salty snacks, etc.) [9]*.* Rundle et al. hypothesized that people, especially children or adolescents, would fare worse concerning weight control at home than when engaged in their usual school curriculum [10]*.* School or working environments provide structure and routine for mealtimes, PA, and sleep schedules, the predominant lifestyle factors involved in obesity risk. Unlike the general population, PWS patients require permanent supervision to avoid food seeking and prevent uncontrolled eating behavior. Living conditions during the lockdown likely led to robust caregiver supervision, as parents or family were generally at home. Caregivers are particularly aware of the risk of weight gain during increased sedentarism. Indeed, medical-expert teams insist on the need of regular PA from childhood. In many homes, PA programs were set up during the lockdown, although PA levels were lower than usual.

However, one recent study showed the negative psychological impact of strict lockdown measures on psychiatric patients during the COVID-19 epidemic [7]*.* Exacerbations of anger and impulsivity were significantly higher for psychiatric patients than healthy controls. Only 12.9% of PWS patients showed behavioral disorders during the lockdown. Impulsive behavior often arises for individuals with PWS from a misunderstanding of social situations or the occurrence of unpredictable situations*.* It is possible that the lockdown, carried out in a ritualized environment with adapted social interactions and a less stressful context, provided more favourable conditions for PWS subjects.

Only three cases of non-severe COVID-19 were reported (3.5%), although 80.2% of the subjects were living in regions where the estimated prevalence of COVID-19 was high (> 9.0%) [11]*.* The high level of compliance with the total lockdown by the PWS patients was likely a contributing factor.

Our study had several limitations, including the fact that our data were acquired in a sample from caregivers’ reports and that no quantitative measures of activity levels or food consumption were available. It is possible that parents’ sense of their children’s behavior was heightened during the lockdown, potentially inadvertently biasing their responses. Moreover, we conducted a single-center study, which might not be representative of the general PWS population.

## Conclusions

In conclusion, the COVID-19 pandemic and lockdown did not have a negative impact on most French adult patients with PWS, despite their well-known psychological vulnerability and susceptibility to gain weight. Favorable changes in lifestyle behaviors during the lockdown, strictly supervised by caregivers in a quiet and reassuring environment, were observed, in parallel with modest weight loss for half of the patients.

## Methods

### Study rationale and design

Eighty-five adults with genetically confirmed PWS followed at the Reference Center for Rare Diseases in Pitié-Salpêtrière Hospital, Paris, France, were included in this monocentric study. The PWS diagnosis was genetically confirmed using routine genetic laboratory methods. First, we performed DNA-based methylation testing to detect the absence of the paternally contributed PWS/AS (Prader Willi Syndrome/Angelman Syndrome) region on chromosome 15q11-q13 at locus *SNRPN* (Small Nuclear Ribonucleoprotein Polypeptide N) with two methods described previously [12–14]. Secondly, molecular mechanism was clarified using a standard fluorescence in situ hybridization (FISH) (deletion). If no deletion was found, analysis of parental and proband DNA with microsatellites was performed to confirm maternal uniparental disomy (UPD).

Telephone interviews were conducted between 15 April and 15 May 2020 by a physician specialized in the care of PWS patients. In France, the complete lockdown lasted from 17 March until 11 May 2020. Patients completed a detailed clinical questionnaire with the help of their caregivers (parents or social workers). The questionnaire included 32 questions related to living conditions during the lockdown, physical and daily activity, sedentary time (time spent seated or lying down when awake and time spent in front of a screen), time sleeping, eating habits (access to food, frequency of eating), medical and behavioral issues (need of medical contact, COVID-19 symptoms in patients and their relatives, frequency of outbursts, anxiety). Weight data before the lockdown were collected from the medical report and weight at the end of lockdown was measured by the patient with the help of the caregiver. Patients and their caregivers provided informed consent.

### Statistical methods

Descriptive statistics of participant characteristics are expressed as the means and standard deviation (SD) for continuous variables and frequencies and percentages for categorical variables. The mean values of patients who lost weight were compared to those who gained weight using two-sample Fisher test for categorical variables, and two-sample Mann–Whitney test for continuous variables. All statistical analyses were performed using GraphPad Prism version 8.0 and statistical significance was considered for two-sided *p*-values < 0.05.

## Data Availability

H. Mosbah and C. Poitou have stored all the data, which are available upon reasonable request.
